# Clinical Parameters vs Cytokine Profiles as Predictive Markers of IgE-Mediated Allergy in Young Children

**DOI:** 10.1371/journal.pone.0132753

**Published:** 2015-07-27

**Authors:** Catherine Lombard, Floriane André, Jérôme Paul, Catherine Wanty, Olivier Vosters, Pierre Bernard, Charles Pilette, Pierre Dupont, Etienne M. Sokal, Françoise Smets

**Affiliations:** 1 Laboratory of Pediatric Hepatology and Cell Therapy, Institut de Recherche Expérimentale et Clinique (IREC), Université Catholique de Louvain, Brussels, Belgium; 2 Machine Learning Group, ICTEAM institute, INGI Department, Louvain School of Engineering, Louvain-la-Neuve, Belgium; 3 Paediatric Gastroenterology and Hepatology unit, Cliniques Universitaires Saint-Luc, Brussels, Belgium; 4 IRIBHM, Université Libre de Bruxelles, Brussels, Belgium; 5 Cliniques Universitaires Saint-Luc, Obstetric unit, Brussels, Belgium; 6 Pole Pneumology, ENT and Dermatology, IREC, Université Catholique de Louvain, Cliniques Universitaires St-Luc, Pneumology Department and Center for Allergy, Brussels, Belgium; Cincinnati Children's Hospital Medical center, UNITED STATES

## Abstract

**Background:**

Allergy afflicts one third of children, negatively impacting their quality of life and generating a significant socio-economic burden. To this day, this disorder remains difficult to diagnose early in young patients, with no predictive test available.

**Objective:**

This study was designed to correlate cytokine profiles with clinical phenotypes of allergy development.

**Methods:**

Three hundred patients were recruited and followed from birth to 18 months of age. They were given a clinical exam at birth and at 2, 6, 12, and 18 months of age, with skin prick tests at 6, and 18 months, in order to have a record of their medical history and determine their allergic status. In addition, mononuclear cells from 131 patients were isolated from cord blood and from peripheral blood samples at 2, 6 and 18 months of age, to analyse their cytokine and chemokine production.

**Results:**

Cord blood mononuclear cells (CBMCs) from future Immunoglobulin (Ig) E-mediated allergic children produced significantly less Interleukin (IL)-12p70 and IL-15 than cells from the rest of the cohort. Multivariate analyses revealed that the best predictive model of allergy was built on cytokine data, whereas the best predictive model of IgE-mediated allergy was built on clinical parameters.

**Conclusions and clinical relevance:**

Although univariate analyses can yield interesting information regarding the immune responses of allergic children, finding predictive markers of the disorder will likely rely on monitoring multiple parameters. Nonetheless these analyses suggest a potential key role for IL-15 in the development of atopic disease. In addition, the study highlights the importance of clinical parameters in predicting the development of IgE-mediated allergy.

## Introduction

Allergic disorders currently affect one third of children, hampering their quality of life and generating a significant cost to their families and society. Although tremendous efforts have been dedicated to understanding the mechanisms of the disease, current treatments are still more therapeutic than preventive. Unlike adults, allergic symptoms in infants are often IgE-independent, making it difficult to diagnose the disorder early. However, early IgE-mediated allergy can lead more commonly to the atopic march with persisting allergy later in life. More studies are therefore needed to unravel the early signs of allergy and to determine whether these could be used to identify children at risk of developing the disease.

The development of allergy has long been linked to an imbalance between T helper (Th)2 and Th1 cytokines, ([1] for review). However, recent studies have implied that the mechanisms involved may be more complex. For example, IL-10 has been shown to inhibit both Th1 and Th2 responses in humans[2]. In addition, a third population of helper T cells, producing IL-17 (Th17 cells), has recently been characterized[3–5]. Although these cells were initially studied for their involvement in autoimmune and inflammatory disorders, recent reports have suggested their role in allergic disorders ([6] for review). Clearly, analysis of cytokine production by cord blood and peripheral blood monocytes could be a key factor in predicting allergy development, but more research is needed to understand the exact role played by each cytokine and its predictive value.

In this study, we followed a cohort of 300 children (CRISTALL cohort) from birth to eighteen months of age in order to unravel potential predictive markers of allergy and/or atopic disease.

## Methods

### Study population follow-up

This study is a monocentric study approved by the Hospital’s ethics committee (comité d’éthique biomédicale hospital-facultaire des Cliniques Universitaires St Luc; ref 2005/20sept/147). Participants were recruited prospectively during a prenatal consultation. Participation in the study was offered to mothers of full-term babies (37 weeks and above) with no significant antenatal pathology and written informed consent was obtained from both parents before inclusion.

Detailed anamnesis (familial, personal, environmental) and clinical examination were performed at 0, 2, 6, 12 and 18 months. Blood samplings were obtained at 0, 2, 6 and 18 months, with skin prick tests at 6 and 18 months for dermatophagoides pteronyssinus and farinae, mix of 5 grasses pollens, cat, dog, aspergillus mix, cow’s milk, white egg, soy and peanut (S1 Table). In case of symptoms related to another allergen, this allergen was added to the skin prick tests. The “allergic-non allergic” classification of the children was performed at 18 months, by two separate paediatricians expert in the field, according to the accepted definitions (Table 1). Children with strong signs of allergy who did not fulfil the criteria were classified as “uncertain”. Allergic children with detectable plasma levels of allergen-specific IgE and/or positive skin prick tests related to their symptoms were considered “IgE-mediated allergic”. The remaining allergic patients were considered “non-IgE-mediated allergic”.

**Table 1:** Definitions of allergy used for the classification of children.

Type of allergy	Criteria	References
**Cutaneous**	Classical atopic dermatitis with at least 1 other criteria: 1) clearly influenced by the diet 2) with bronchial hyperreactivity 3) with positive skin prick test 4) with first degree familial history of atopy	[30, 31]
**Respiratory**	Minimum 3 wheezing episodes in the past year with 1 major criteria or 2 minor criteria: 1) major: asthma in parents, environmental tobacco, medical diagnosis of atopic dermatitis or food allergy 2) minor: positive IgE or skin prick test, wheezing outside RSV infection, eosinophilia	[32–34]
**Food allergy**	Symptoms driven by one or several food, not present under eviction diet and recurring after provocation	[35, 36]

### CBMC/PBMC isolation

Cord blood mononuclear cells (CBMCs) or Peripheral blood mononuclear cells (PBMCs) were isolated by density gradient centrifugation using Ficoll-Paque (GE Healthcare, Diegem, Belgium) and resuspended in RPMI-1640 supplemented with 1% of Penicillin / Streptomycin, 2 mM L-glutamine, 1% non-essential amino acids (Invitrogen,) and 10% FCS (A&E Scientific, Marcq, Belgium) (complete RPMI). Cell viability as estimated by trypan blue (Sigma Aldrich, Bornem, Belgium) dye exclusion was found to be >95%.

### Cell stimulation for cytokine quantification

CBMCs/PBMCs (5 x 10^5^/well in a 48-well plate) were cultured in 500 μl of complete RPMI at 37°C under a humidified atmosphere containing 5% CO_2_. Cells were left unstimulated or stimulated with phytohemagglutinin (PHA) (5 μg/ml) or Lipopolysaccharide (LPS) (1 μg/ml) (Sigma Aldrich), for 24 and 96 hours. Culture supernatants were then harvested, centrifuged at 5000 rpm for 5 min and stored at -20°C until use.

### Determination of plasma total and allergen-specific antibody response

Plasma was obtained by blood centrifugation at 1800xg for 15mn, followed by centrifugation at 4000 rpm for 10mn. The samples were then frozen at -20°C until use. Total IgE and specific-IgE responses to common respiratory (Dermatophagoides pteronyssinus (Der p), cat dander (Fel d), Phleum pratense (Phl p), Birch pollen (Bet v)) and food allergens (peanut, cow’s milk β-lactoglobulin, egg white) were assayed using the ImmunoCap system (Phadia, Uppsala, Sweden), according to the manufacturer’s protocol (detection limit 0.1kU/l). Allergen-specific IgE levels above 0.1kU/l were considered as positive.

### Cytokine quantification

Cytokines and chemokines were measured in culture supernatants by Luminex technology (Biorad) using a commercial 27-plex kit, and the data were analyzed using the Bio-plex manager software (Biorad). Culture supernatants from adult PBMCs stimulated with PHA were used as a positive control on each plate. Cytokines for which the positive control showed too much inter-plate variation (ratio of the maximum control values over the minimum control values above five after removal of the outliers) or the test values were generally out of range were excluded from the analysis. In total, 9 cytokines were filtered out due to this criterion. The remaining cytokines and their respective limit of detection are shown in Table 2. The cytokine concentration in each sample was obtained by subtracting the value obtained in the non-stimulated sample from each stimulated sample.

**Table 2:** Cytokines and chemokines analysed by Luminex and detection limits.

Cytokine	Detection limit (pg/ml)	Inter-plate variability (ratio max/min)
IL-1 β	0,65	3.94
IL-1ra	1,21	2.29
IL-2	0,35	1.60
IL-4	0,08	1.48
IL-7	0,71	1.56
IL-9	0,38	1.40
IL-10	0,52	1.62
IL-12p70	0,64	1.37
IL-15	0,49	1.54
IL-17	0,53	1.59
Eotaxin	0,44	2.2
FGF	0,3	1.80
G-CSF	0,44	2.05
GM-CSF	0,18	1.72
IFN-γ	0,55	2.38
IP-10	0,75	2.37
TNF-α	1,6	1.50
VEGF	0,69	1.84

### Statistical analysis

Results are expressed as mean ± standard deviation (SD) or as median and range. Univariate analyses were performed as follows: continuous variables were compared with the unpaired t-test; discrete ordinal variables were compared with the Mann-Whitney test; discrete binary and nominal variables were compared with the Chi-square test (or Fisher’s exact test when appropriate).

A second set of analyses were conducted on cytokine data and 8 clinical variables a priori believed to be more discriminant (number of allergic first degree relatives, tobacco exposure of the mother during pregnancy, exclusive breastfeeding at birth, 2, 6 and 12 month visits, delivery mode, and presence of pets during pregnancy). Univariate analyses, descriptive of the observed patients, were performed with the objective of ranking. Considering the large number of variables (296 after cleaning of the data), the reported p-values of the tests were corrected for multiple testing using the Benjamini-Hochberg correction[7]. This correction takes into account all the 296 variables. There are 288 cytokine measurements: 18 cytokines in two different stimulations (LPS, PHA) for two stimulation durations (24h, 96h) for four time points (CB, 2, 6 and 18 months). We also include the 8 clinical factors in the correction. In addition to univariate analyses, multivariate analyses were performed on cytokine data or cytokine and clinical data to try and determine the best predictive model of allergy and/or IgE-mediated allergy development. As the objective of those analyses was to classify new patients, a resampling protocol [8, 9] was used. This procedure allows to estimate the performances of predictive models on new, previously unseen data. To do so, it repeatedly learns predictive models on subsets of the dataset and assesses the classification performances on the remaining data that were not used during the learning part. The reported metric is the Balanced Classification Rate (average between Specificity and Sensitivity). The experimental setting was as follows:

Repeat 200 times:

- Sample 90% of the cohort as training set
  ∘ Rank features according to T-test and Fischer’s exact test
  ∘ Learn a predictive model from the *s* best ranked variables
- Classify on 10% unseen data
- Record Balanced Classification Rate (BCR)

So as to provide a global performance measure, BCRs are averaged over all samplings. The 95% confidence interval is computed with Nadeau’s corrected statistics for resampling protocols[10].

Because not all features are expected to be relevant for the prediction task, the multivariate analyses consider subsets of the 296 variables. Predictive models are built for several signature sizes *s* i.e. considering only the *s* best variables of a univariate ranking [9] (with a t-test and a Fisher's exact test). Note that the variables taken into account in the predictive models are not limited to those which are statistically significant. Indeed, multivariate approaches benefit from feature interactions that cannot be highlighted by univariate statistical tests. While the rankings are computed from 90% data in the experimental protocol, they are very close to the global variable ranking of the univariate analysis. To be able to take into account clinical variables, three classifiers were used:

- Random Forest[11] which is an ensemble of randomized decision trees
- SVM[12] which performs non-linear classification using a linear kernel for cytokine features and a clinical kernel[13] for clinical variables
- 1-Nearest Neighbour[14] which classifies new samples according to the nearest training instance

It is important to note that multivariate analyses tackle a much harder problem than just describing the data as in univariate analyses since the goal is to make prediction on new data samples. Moreover, missing values are more problematic in multivariate analyses. While keeping only samples for which there is no missing value is possible for univariate tests, doing the same for multivariate analyses would lead to elimination of all samples. Hence, we resort on imputation for the features with more than 50% available data, inferring missing values based on similar data. For each variable, the missing values of the training set are replaced by the average or statistical mode of the samples of the same class. In order to avoid an optimistic evaluation of the predictive performances, the missing values of the remaining 10% data are replaced by the global average or statistical mode of the variable, computed on the training set.

## Results

### Demographic characteristics

**Fig 1:**
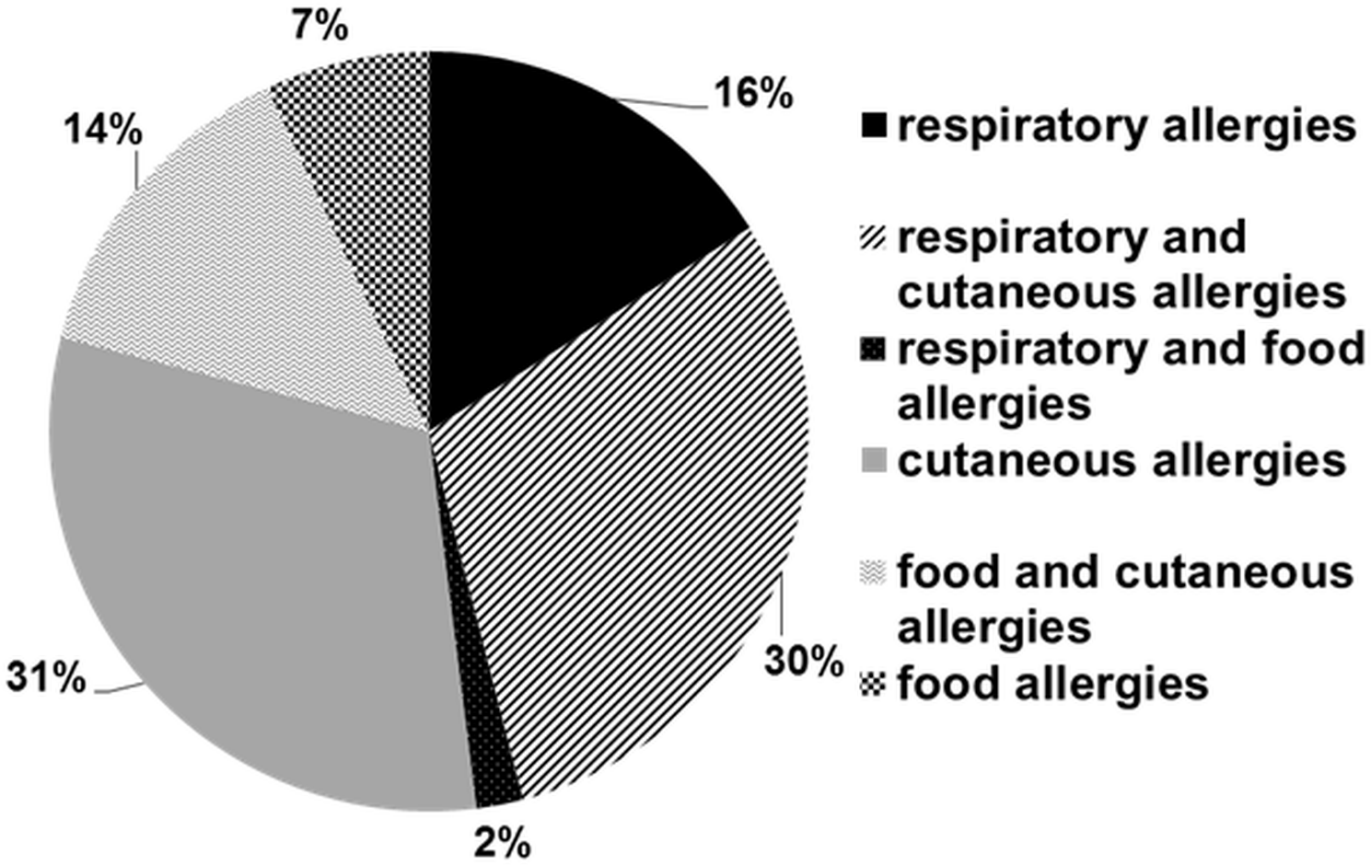
Demographic information on the cohort. Types of allergies diagnosed in the cohort up to eighteen months and the corresponding percentages of patients affected.

A total of 297 patients were effectively enrolled in the study, out of which 221 were still actively participating at 18 months, corresponding to a 25.59% dropout rate. Based on the information collected over the five check-up visits, 141 children (67%) were classified as “non-allergic”, 43 (21%) were classified as “allergic”, and 25 (12%) were labelled as “uncertain”. The remaining 12 children were not seen at 18 months and therefore could not be classified. Among the allergic children, 37% (16 patients) were atopic. Interestingly, the sex of the child had no influence on his/her propensity to develop allergies (p>0.05). The median age at diagnosis of allergy was 6 months, ranging from 1.5 to 18 months. The predominant type of allergy was cutaneous allergies (atopic dermatitis and/or allergic urticaria), alone (31%) or in association with respiratory allergies (bronchial hyperreactivity, 28%) (Fig 1 and Table 1). The development of allergy did not appear to have an impact on the overall physical development of the child (p>0.05 for height and weight, data not shown).

### Clinical data

#### Family history

The CRISTALL study confirmed the importance of family history, with an impact of the number of allergies diagnosed in first degree parents on the development of allergies in the child (0.88 ± 0.84 in the non-allergic group vs 1.37 ± 0.87 in the allergic group; p = 0.008) and 90.7% of allergic children versus 61.7% of non-allergic children having at least one risk factor among the first degree relatives (p = 0.008). Having an allergic mother seemed to be more strongly correlated with the development of allergies in the child than having an allergic father, although the data did not reach statistical significance, probably due to the small sample size (p>0.05).

#### Environmental factors

Several environmental factors were studied in order to determine their potential impact on the development of allergy. Following the check-up visit at birth, we found that 17% of non-allergic children had had exposure to dogs during pregnancy, whereas none of the allergic children had had any contact (p = 0.03). However we found no statistical difference between allergic and non-allergic children’s exposure to pets during the follow-up visits.

#### Introduction of solid food

We found a lower consumption of regular baby formula among the allergic patients (50.5% of non-allergic vs 22.5% of allergic children at 6 months, p = 0.0083; 59.1% vs 24.4% at 12 months, and 70% vs 35.1% at 18 months, p = 0.003), who in turn consumed more hydrolysed and soy formula (59.1% of allergic vs 24.4% of non allergic at 12 months, and 70% vs 35.1% at 18 months, p< 0.0025), as well as a tendency to delay the introduction of certain solid foods in their diet.

#### Other risk factors

We found no significant impact of birth by caesarean section, with 27.9% of allergic patients versus 19.1% of non-allergic patients born by caesarean section (p>0.05). Exclusive breastfeeding seemed to last longer for non-allergic patients (14.5% of non-allergic patients vs 7.1% of allergic patients at 6 months of age, and 6.0% vs 0% at 12 months of age), suggesting a beneficial effect of breastfeeding, but the differences seen were not statistically significant (p>0.05).

#### Skin prick test and plasma IgE levels

**Fig 2:**
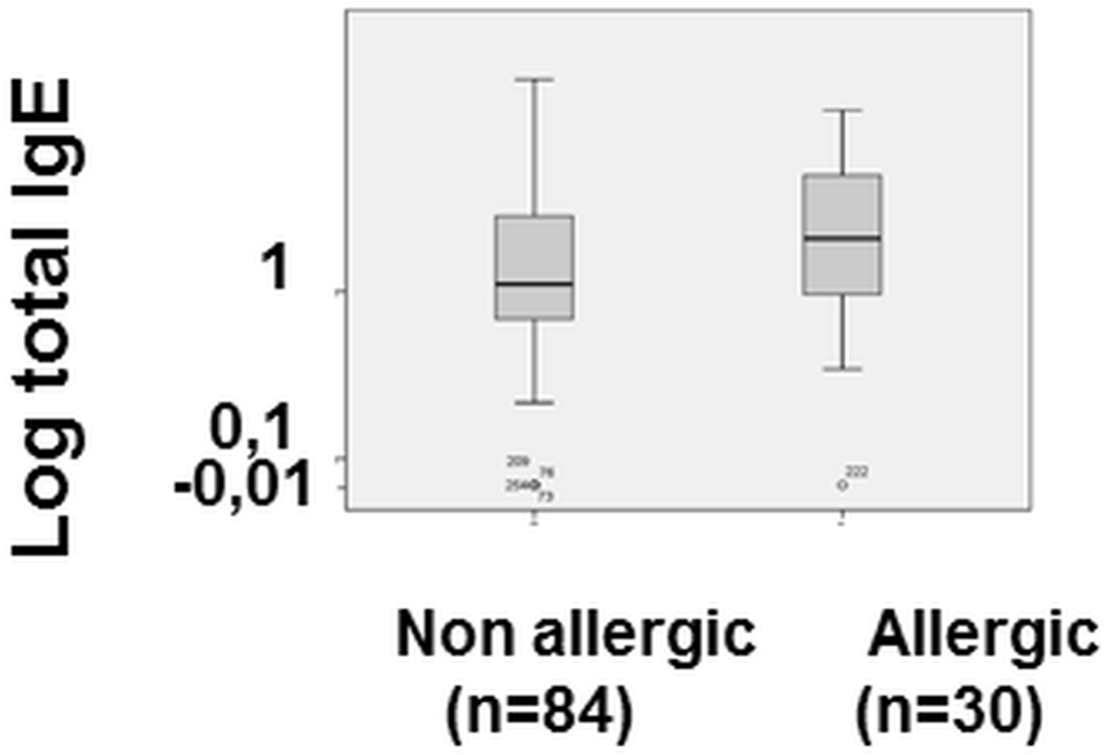
Total IgE concentration in the plasma of 18 month old allergic and non-allergic children. Plasma collected during the 18 month check-up visit was assayed for total IgE. The data are shown as box plots of the median, lower and upper quartile ± standard deviation from the median. Individual dots represent outliers.

Although a higher incidence of positive skin prick tests in the allergic population was expected since it was one of the potential criteria used to classify the patients as allergic, it was interesting to see that, at 6 months of age, before eggs had been usually introduced into the diet, the skin prick test for egg white was positive in 12.2% of allergic patients, versus 1.5% of non-allergic patients (p = 0.05). At 18 months of age, allergic children had a higher frequency of positive skin prick test results to egg (16.2% vs 2%; p = 0.03), and cow milk (13.5% vs 0%; p = 0.008), confirming the development of atopic disease in that population. It is interesting to note that the percentage of allergic children showing a positive reaction to these allergens remained fairly low. Total IgE levels had a tendency to be higher in the allergic group than the non-allergic group at 18 months of age although the difference was not statistically significant, (30.9 vs 15.58; p = 0.154) (Fig 2), confirming the development of one or more sensitizations. The percentage of children with positive **radioallergosorbent tests** (RAST) at 18 months remained low despite being higher in the allergic group, highlighting the difficulty in diagnosing allergy based on this parameter (25% of allergic children vs 13.75% of non-allergic children with RAST>0.1kU/l, and 22% of allergic children vs 3.75% of non-allergic children with RAST>0.35kU/l) (data not shown).

### Cytokine production

In order to study a potential correlation between cytokine production and the development of allergy, we measured the production of various cytokines by CBMCs or PBMCs following a non-specific stimulation with LPS or PHA on a reduced set of patients (15 IgE-mediated allergic, 30 non-IgE-mediated allergic or uncertain and 86 non-allergic). It has to be noted that we analysed at the most 74 patients per condition due to missing values. The data are presented as differences between the stimulated samples and the non-stimulated samples for both allergic and non-allergic children.

The classification of the patients into allergic and non-allergic groups did not yield any significant difference in the cytokine production after correction for multiple testing, despite some interesting trends, particularly for cytokines produced by CBMCs (Table 3: five best ranked cytokines). However, CBMCs from future IgE-mediated allergic children produced significantly less IL-12p70 and IL-15 following stimulation than those of the rest of the cohort (non-IgE-mediated allergic and non-allergic children, Table 4). At 6 months of age, PBMCs from future IgE-mediated allergic children produced less IL-1β following stimulation. Finally, by 18 months, PBMCs from IgE-mediated allergic children produced less **vascular endothelial growth factor** (VEGF) following stimulation than the other children.

**Table 3:** Comparison of the cytokine profiles of cord blood mononuclear cells from allergic (All) and non allergic (non all) children.

Cytokine	Stimulation	Difference (cytokine concentration in pg/ml)	Uncorrected p value**	Corrected p value*
**TNF-alpha**	LPS 24h	All (3115,18) **<** non all (6059,83)	0,001171648	0,2180631
	PHA 24h	All (3862,2) **<** non all (6854,98)	0,006945071	0,3426423
**IL-15**	PHA 96h	All (11,12) **<** non all (25,06)	0,0014734	0,2180631
**IL-17**	PHA 96h	All (39,14) **<** non all (73,77)	0,005384663	0,3426423
**FGF**	PHA 96h	All (18,38) **<** non all (35,8)	0,006945451	0,3426423

*Corrected p value: p-value obtained after correction for the large number of variables tested using the Benjamini-Hochberg correction[7]

**Uncorrected p-value: p-value obtained before the correction was applied.

**Table 4:** Comparison of the cytokine profiles of CBMCs and PBMCs from IgE-mediated allergic children (IgE+) and the rest of the cohort (IgE- and non all) over the first eighteen months of life.

Time of blood sampling	Cytokine	stimulation	Difference (cytokine concentration in pg/ml)	Corrected p value*
**cord blood**	**IL-12p70**	LPS 96h	IgE+ (0,71) **<** IgE- and non all (2,34)	0,02642649
	**IL-15**	PHA 24h	IgE+ (5,30) **<** IgE- and non-all (19,21)	0,007204243
		PHA 96h	IgE+ (3,74) **<** IgE- and non-all (21,87)	0,02108183
**6 months**	**IL-1beta**	LPS 24h	IgE+ (290,36) **<** IgE- and non-all (1374,43)	0,01008838
		LPS 96h	IgE+ (183,17) **<** IgE- and non-all (847,64)	0,04729766
**18 months**	**VEGF**	LPS 96h	IgE+(1,57) **<** IgE- and non-all (93)	0,04729766

*Corrected p value: p-value obtained after correction for the large number of variables tested using the Benjamini-Hochberg correction[7].

### Predictive models

Multivariate analyses were performed using cytokine data and/or clinical data (see materials and methods for details) to try and determine the best predictive models of allergy (both IgE-mediated and non-IgE-mediated allergic children) and/or IgE-mediated allergy development. The best predictive model of allergy (BCR = 69.2%; 95%CI: 56%; 84.8%) was obtained from a non linear SVM[12] built from *s* = 4 cytokine data obtained with CBMCs. The cytokines that appeared most often in the signatures were the best ranked cytokines from the univariate analyses (Table 3). The two other classifiers performed worse, although not significantly due to the large confidence intervals. On the other hand, the best predictive model of IgE-mediated allergy (BCR 73%; 95%CI: 56.9%; 89%) was obtained from a 1-Nearest Neighbour[14] based on clinical parameters only (number of allergic first degree relatives, tobacco exposure of the mother during pregnancy, exclusive breastfeeding at 2, 6 and 12 month visits, delivery mode, and presence of pets during pregnancy). Again, the other classifiers performed worse but the differences were not significant. Finally, it has to be noted that the 95% CI for these models are fairly large and the BCRs could still be improved.

## Discussion

In this study, we have prospectively followed a large panel of cytokines in infants from birth to 18 months, and correlated them with their allergic status. Although differences in the cytokine profiles of allergic and non-allergic children could be detected as early as birth, the differences seen were not statistically significant when corrected for multiple testing. Focusing on some cytokines could have been interesting in this context but, because of the young age of the subjects, limited amounts of sample were available and we chose to use the multiplex technique. It is however interesting to note that the best predictive model of allergy remained the model based on cytokine production by CBMCs, highlighting the fact that these variables are better markers of allergy as a group than taken individually.

Although we have measured total and allergen-specific IgEs at earlier timepoints, we only found total IgEs to be higher in allergic children at 18 months, and only in about 30% of all cases. Similarly, skin prick tests were only positive in a small number of allergic children, even at 18 months of age. This is consistent with reports that most allergies in young children are IgE-independent and cannot be detected with skin prick tests and/or allergen-specific IgEs, highlighting the need for better testing methods[15–18]. It is also in line with a study showing no correlation between cord blood IgE levels and the development of asthma later in life[19]. However, for ethical reasons, we had to limit the number of tests performed and we could have underestimated IgE-positive allergy by missing some sensitization.

The cut-off value for allergen-specific IgEs to be used in determining a young child’s atopic status remains a matter of debate. Physicians traditionally used a cut-off of 0.2 kU/l for newborns and 0.35 kU/l for infants, but are currently moving towards lowering the cut-off to 0.1kU/l. We decided to use the lowest cut-off so as to not miss any potential candidate within the allergic children because of their very young age. In this group, the specificity of the 2 cut-offs did not significantly differ.

Segregating patients into IgE-mediated and non-IgE-mediated allergic with non-allergic for the cytokine analyses yielded significant differences: CBMCs from future IgE-mediated allergic children were found to produce lower levels of IL-15 and IL-12p70 following non-specific stimulation than those from the other children. Similarly, PBMCs from IgE-mediated allergic patients produced less IL-1β (cytokine involved in the priming of T cells towards Th17) at 6 months following LPS stimulation.

IL-15 was initially described as a T cell growth factor. It has been shown to play a role in the development of Th17 cells in the context of autoimmune and inflammatory disorders such as rheumatoid arthritis and celiac disease[20–22]. In addition IL-15 is known to promote the proliferation and survival of CD8+ memory T cells[23, 24]. Although the precise role of IL-15 in allergy is not completely understood yet, studies have shown that this cytokine can prevent the development of allergic rhinitis[25].

IL-12 has been known to induce the production of IFN-γ and reduce IL-4 induced IgE production[26, 27]. In this study, CBMCs from future IgE-mediated allergic children produced less IL-12p70. These results are consistent with previous reports that low numbers of IL-12 and IFN-γ producing CBMCs is linked to early childhood IgE sensitization[28]. Interestingly, despite a lower production of IL-12p70 at birth, future IgE-mediated allergic children did not start producing IgEs until 6 months of age.

It is noteworthy that these cytokines not only received the best ranking in the univariate analyses but also appeared in the majority of the signatures built through multivariate analysis, highlighting their importance in the development of the disease.

From a clinical point of view, our study confirms previously reported data concerning the demographics of allergy and the influence of family history on the development of allergies.

Moreover, we found a higher number of non-allergic children among the children who had been exposed to dogs during pregnancy, further suggesting that dog ownership could have a protective effect on sensitization to aeroallergens ([29] for review).

Some of the clinical data obtained, however, must be considered with caution. Indeed, we found a higher consumption of hydrolysed or soy milk and a tendency to delay the introduction of solid food in the allergic group between 12 and 18 months of age, which could suggest a potential protective effect of exposure to allergens. However, it is difficult to tell for sure whether the lack of exposure to allergens has played a role in the development of allergies, or whether it merely represents precautions put in place as a result of a significant family history of allergies in children who would likely become allergic, as is often recommended.

It is interesting to note that the best predictive model of IgE-mediated allergy was based on clinical data only despite significant differences in the cytokine production by CBMCs. However, one should not be surprised by the apparent discrepancy between univariate and multivariate analyses considering that univariate analyses are descriptive of a given group of patients whereas multivariate analyses use models based on a given group of patients to predict the status of future patients. Indeed, data with a good descriptive value do not necessarily warrant a good predictive value.

Together these results suggest that despite clear differences in the cytokine profiles of IgE-mediated and non-IgE-mediated allergic infants, clinical parameters remain the most relevant in predicting disease development at 18 months.

Finally, it has to be noted that the allergic group studied was fairly heterogeneous since we included all types of allergies in the analysis knowing that some of these will probably disappear later in life. Further studies are underway to determine whether cytokine data can better predict the allergic status of the children at 3 and 5 years of age.

## Supporting Information

S1 Table Reagents used for the skin prick tests and references. This Table presents the various allergens used to perform the skin prick test as well as the corresponding reference number from the supplier.
(DOCX)
