# Supplementary material for: Clinical Parameters vs Cytokine Profiles as Predictive Markers of IgE-Mediated Allergy in Young Children
Source: PLoS One. 2015 Jul 27;10(7):e0132753. doi: 10.1371/journal.pone.0132753 (PMC4516234; doi:10.1371/journal.pone.0132753)
Supplement: S1 Table — This Table presents the various allergens used to perform the skin prick test as well as the corresponding reference number from the supplier. (DOCX) [file pone.0132753.s001.docx]

**S1 Table. Reagents used for the skin prick tests and references**

| **Reagent used for the skin prick test** | **Reference** | **Supplier** |
| --- | --- | --- |
| histamine HCL (positive control) | 2077 | Stallergenes (France) |
| negative control | 2069 |  |
| Dermatopagoides pteronyssinus | 315 |  |
| Dermatophagoides farinae | 314 |  |
| 5 grasses mix | 688 |  |
| dog hair | 509 |  |
| cat hair | 507 |  |
| aspergillus mix | 401 |  |
| peanut | 109 |  |
| egg white | 143 |  |
| soy | 190 |  |
| cow milk | N/A | N/A |
